# Hyperandrogenism-Insulin Resistance-Acanthosis Nigricans Syndrome

**DOI:** 10.1155/2015/193097

**Published:** 2015-07-02

**Authors:** A. H. Dédjan, A. Chadli, S. El Aziz, A. Farouqi

**Affiliations:** Endocrinology, Diabetology and Metabolic Diseases, Ibn Rochd University Hospital Center of Casablanca, 20360 Casablanca, Morocco

## Abstract

*Introduction*. Female hyperandrogenism is a frequent motive of consultation. It is revealed by hirsutism, acne or seborrhea, and disorders in menstruation cycle combined or not with virilisation signs. Several etiologies are incriminated but the hyperandrogenism-insulin resistance-acanthosis nigricans syndrome is rare.* Observation*. A 20-year-old girl, having had a five-year-old secondary amenorrhea. The exam revealed a patient, normotensive with a body mass index at 30 kg/m^2^ and a waist measurement of 120 cm, a severe hirsutism assessed to be 29 according to Ferriman Gallwey scale, virilisation signs of male morphotype, clitoridic hypertrophy and frontal alopecia, and an acanthosis nigricans behind the neck, in the armpits and elbows. The assessment carried out revealed testosteronemia at 1.28 ng/mL, which is more than twice the upper norm of the laboratory. Imaging studies were negative for both ovarian and adrenal masses. The retained diagnosis is HAIR-AN syndrome probably related to ovarian hyperthecosis and she was provided with androcur 50 mg/day and estradiol pills 2 mg/day and under hygiene-dietetic conditions.* Conclusion*. This case proves that HAIR-AN syndrome could be responsible for severe hyperandrogenism with virilisation signs. It must be retained after discarding the tumoral causes and when there are signs of insulin resistance.

## 1. Introduction

Female hyperandrogenism is a frequent motive of consultation in endocrinology, in dermatology, or in gynecology. It is revealed by hirsutism, acne or seborrhea, and disorders in menstruation cycle combined or not to virilisation signs that are androgenic alopecia, harsh voice, hyperhidrosis, clitoromegaly, and/or big lips. Hirsutism, being the most common symptom, is found in approximately 5% of women in procreation age [1]. The etiologies of the hyperandrogenism are dominated by polymicrocystic ovaries (71–86%); congenital hyperplasia of the adrenal (3–10%); the tumoral ovarian and adrenalian causes (0.3%); and idiopathic hirsutism (10%) [2, 3]. The hyperandrogenism-insulin resistance-acanthosis nigricans syndrome (HAIR-AN syndrome) is also incriminated [4, 5].

We here describe the case of a young girl presenting the HAIR-AN syndrome.

## 2. Observation

**Figure 1:**
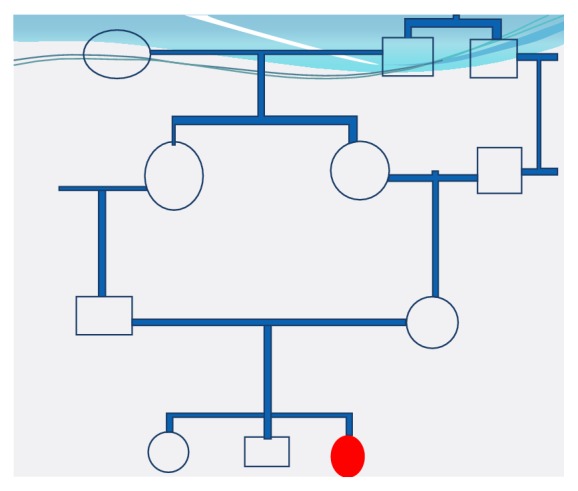
Genealogical tree of the family.

A 20-year-old girl have had her first menstruation at the age of 14 years, was single hospitalized for hirsutism with secondary amenorrhea, and was evoluting for 5 years with no abdominal pain and no pelvic heaviness. The antecedents were mental retardation and as family antecedents consanguine marriage of her parents (Figure 1), a brother and a sister suffering mental retardation, and her mother being obese, diabetic, and hypertensive. There was no antecedent of specific drug intake.

**Figure 2:**
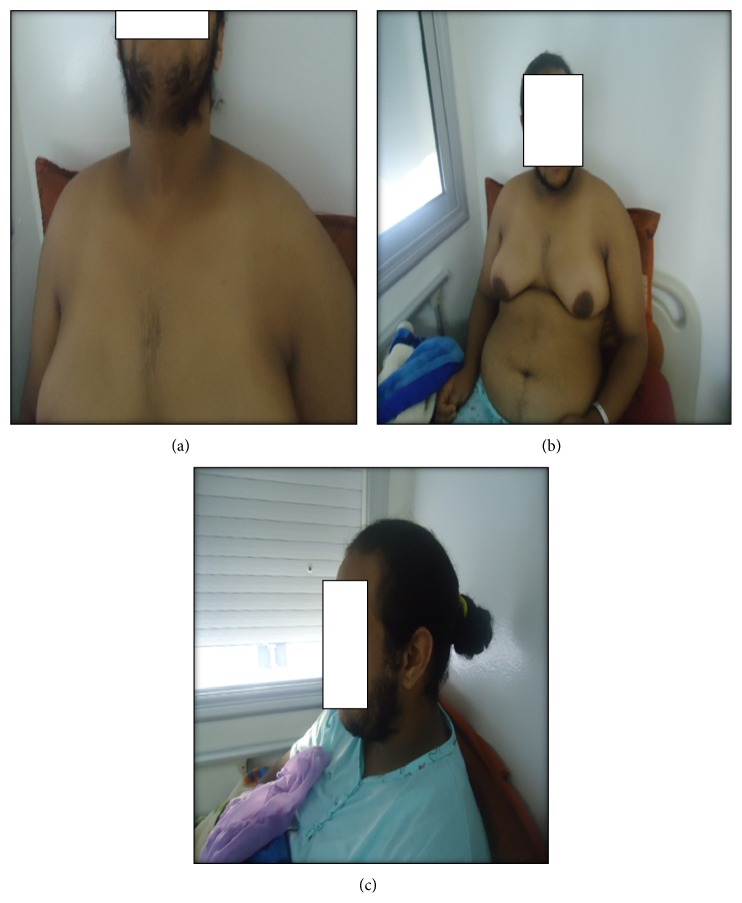
Hirsutism.

**Figure 3:**
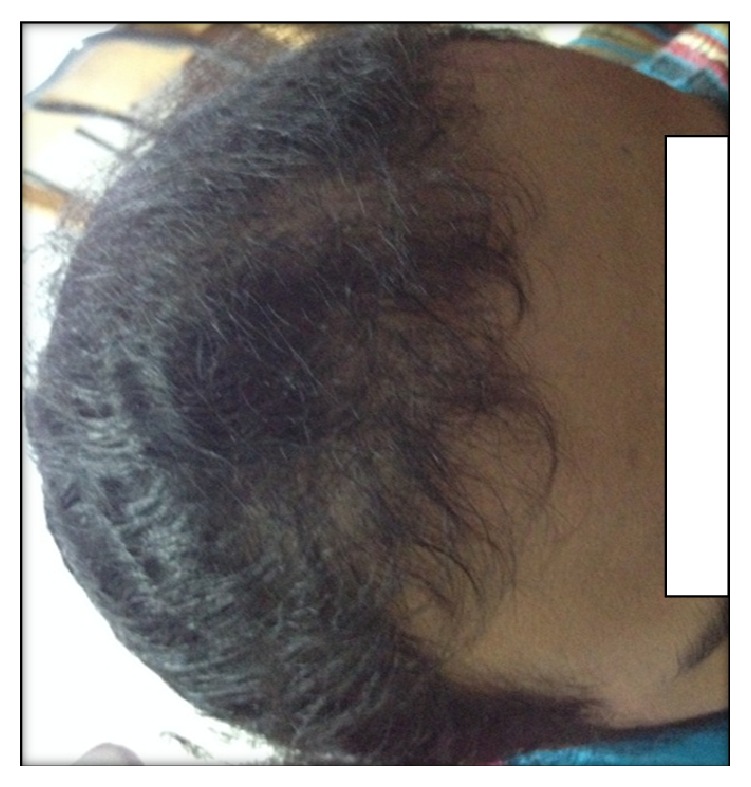
Frontal alopecia.

**Figure 4:**
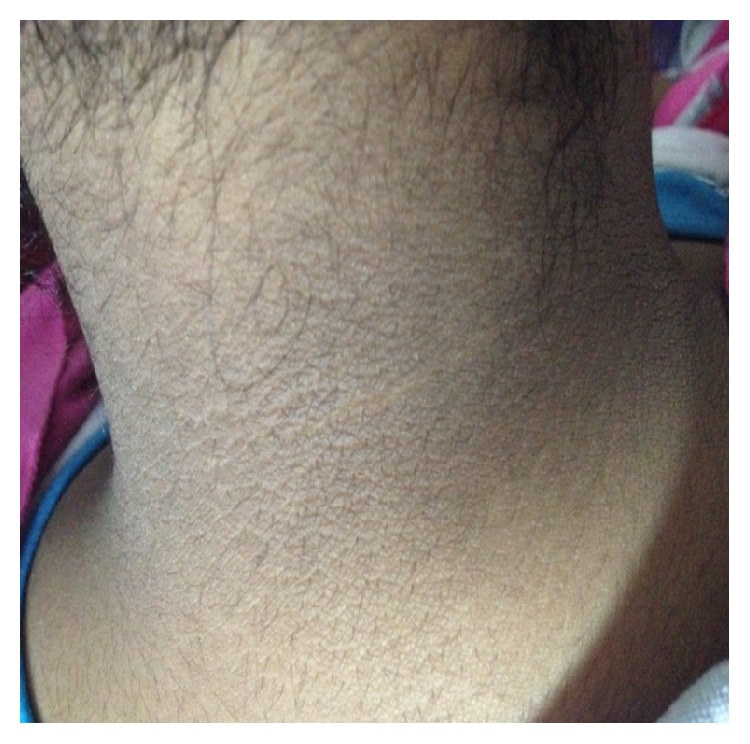
Acanthosis nigricans.

The exam revealed a patient in good general condition, normotensive at 130/86 mmHg with a body mass index (BMI) at 30 kg/m^2^ and a waist measurement of 120 cm, capillary glycemia 1.01 g/L, a severe hirsutism assessed to be 29 according to Ferriman Gallwey scale (Figure 2), inflammatory blackheads on the back and on the chest virilisation signs of male morphotype, clitoridic hypertrophy and frontal alopecia (Figure 3), and an acanthosis nigricans behind the neck (Figure 4), in the armpits and elbows, while there is no galactorrhea, or melanodermia, and she is ranked S5P5 on TANNER scale.

The assessment carried out revealed testosteronemia (MEIA) at 1.28 ng/mL [0.10–0.28] which is more than twice the upper norm of the laboratory, the 17 alpha hydroxyprogesterone at 0.8 ng/mL [0.5–1.1], the dehydroepiandrosterone at 3.5 ng/mL [1–8], the delta-4-androstenedione at 1.5 ng/mL [0.7–3.5], the progesterone at 2 ng/mL [0.35–0.93], the estradiol at 121 pg/mL [39–189], the ACTH at 9.6 pmol/L [1.6–13.9], the *β*hcg < 2 ui/L to discard pregnancy, the anti-Müllerian hormone at 33.7 pmol/L [14.28–48.55], the prolactinemia at 5.21 ng/mL [3.34–26.72], the TSHus at 2.63 *μ*UI/mL [0.27–4.2], and the glycated hemoglobin (HbA1C) at 5.2%; the renal assessment was normal; so was the hepatic one whereas the lipidic assessment indicated a low high density lipoprotein at 0.35 g/L and the karyotype was normal (46XX).

Morphologically, pelvic ultrasound revealed normal ovaries (left ovary: 7.0 cm^3^ right ovary: 6.1 cm^3^), while abdominal TDM showed normal adrenal and ovarian MRI detected a normal left ovary whereas, with no identified follicle, the right one remained unseen.

The retained diagnosis is HAIR-AN syndrome probably related to ovarian hyperthecosis. In fact, virilisation-combined clinical hyperandrogenism with testosteronemia which is two times superior to the upper laboratory norm pushes us to think first of an ovarian tumoral or adrenal cause [6]. So the value of the 17 alpha hydroxyprogesterone at 0.8 ng/mL exempts the Synacthen stimulation test because it is lower than 2 ng/mL [3] and excludes the diagnosis for congenital hyperplasia of adrenal. Abdomen TDM did not detect any adrenal mass and the dehydroepiandrosterone (DHEA) which rebecame normal discarded an adrenal tumor. Pelvic ultrasound and pelvic MRI did not detect any tumor or follicles in the ovaries. Moreover, the anti-Müllerian hormone was normal which is in disfavor of SOPK.

Being unable to undergo catheterism of the ovarian vein in our conditions and of annexectomy due to her age, she was provided with androcur 50 mg/day and estradiol pills 2 mg/day (20/28 days) under hygiene-dietetic conditions; she was also advised to have laser depilation.

## 3. Discussion

HAIR-AN is a subset of the polycystic ovary syndrome, in which patients show severe insulin resistance. Both genetic and environmental factors, such as obesity, are related to the development of HAIR-AN. Diagnosis is primarily clinical and laboratory tests may give further support.

HAIR-AN syndrome is found in 1 to 3% in women presenting a hyperandrogenism [5]. In physiopathology, it is established that stromal ovarian cells synthetise androgens when they are stimulated by LH or HCG. We also observed that the steroidogenic activity of these cells was increased by insulin. The latter is a determining element in the severity of hirsutism [7]. There can even exist a stronger correlation with the severity of hirsutism than the level of observed hyperandrogenism [8]. IGF1, proteic molecule having a high degree of homology with insulin, has the same stimulating power of this steroidogenesis.

The maternal antecedent of the metabolic syndrome present in the patient as well as the presence of acanthosis nigricans well express the insulin resistance and could account for hyperandrogenism and the virilisation such as the one also detected in a 12-year-old Vietnamese patient who showed signs of hyperandrogenism and virilisation in relation with hyperinsulinism.

Regarding treatment, to decrease insulin resistance we preferred lifestyle change as insulin-sensitizing agents because we do not have a marketing authorization for this indication. Other choices to decrease ovarian hyperandrogenism, such as estroprogestatif pills, are provided because of their antigonadal action that inhibits LH resulting in lower ovarian androgens; their increasing sex hormone binding globulin is also recognized thus decreasing bioavailable testosterone. In our case the choice of andocur is due to the severity of hyperandrogenism.

Antiandrogens such as spironolactone and flutamide are used elsewhere, but we do not have a marketing authorization. It is the same for the inhibitors of 5*α*-reductase [4].

## 4. Conclusion

This observation proves that HAIR-AN syndrome could be responsible for severe hyperandrogenism with virilisation signs and that testosteronemia two times superior to the laboratory norm does not immediately lead to a tumoral cause. The HAIR-AN syndrome must be retained after discarding the tumoral causes and when there are signs of insulin resistance and of acanthosis nigricans. In our context the diagnosis of the insulin resistance is clinical. We could have confirmed it biologically through the dosing of insulin. Likewise, the oral glucose tolerance tests can be called for. Due to the lack of means, we could not carry them out.
